# The “Great Debate” at Immunotherapy Bridge 2020, December 3rd, 2020

**DOI:** 10.1186/s12967-021-02811-8

**Published:** 2021-04-07

**Authors:** Paolo A. Ascierto, Joshua Brody, Lisa H. Butterfield, Olivera J. Finn, John Goldberg, Francesco Perrone, Ryan J. Sullivan, Bernard A. Fox, Patrick Hwu, Igor Puzanov

**Affiliations:** 1 Department of Melanoma, Cancer Immunotherapy and Innovative Therapy, Istituto Nazionale Tumori IRCCS “Fondazione G. Pascale”, Naples, Italy; 2 Lymphoma Immunotherapy Program, Icahn School of Medicine at Mount Sinai, New York, NY USA; 3 Parker Institute for Cancer Immunotherapy, Microbiology and Immunology, University of California San Francisco, San Francisco, CA USA; 4 Department of Immunology, University of Pittsburgh School of Medicine, Pittsburgh, PA USA; 5 Clinical Development, Oncorus, Cambridge, MA USA; 6 Unit of Clinical Trial, Istituto Nazionale Tumori IRCCS “Fondazione G. Pascale”, Naples, Italy; 7 Hematology-Oncology Dept, Massachusetts General Hospital, Boston, MA USA; 8 Earle A. Chiles Research Institute, Robert W. Franz Cancer Research Center, Providence Cancer Institute, Portland, OR USA; 9 Moffitt Cancer Center, Tampa, FL USA; 10 Department of Medicine, Roswell Park Comprehensive Cancer Center, Buffalo, NY USA

**Keywords:** Immunotherapy, Cancer vaccine, Checkpoint inhibitors, Nivolumab, Pembrolizumab, Overall survival, Progression-free survival, Clinical trials

## Abstract

As part of the 2020 Immunotherapy Bridge virtual congress (December 2nd–3rd, Italy), the Great Debate session featured counterpoint views from leading experts on three clinical questions in immunotherapy today. The first of these was whether antitumoral vaccination is still a treatment option. The second topic debated whether anti-programmed death (PD)-1/PD-ligand (L)1 blockade should be the backbone for immunotherapy combination. Finally, the use of innovative study designs and surrogate endpoints was considered from both an academic and industry perspective. For each topic, two experts presented the argument and counter-argument in support of two different points of view. As with previous Bridge congresses, the debates were assigned by meeting Chairs and positions taken by experts during the debates may not have necessarily reflected their respective personal view. The views summarised in this article are based on available evidence but may reflect personal interpretation of these data, clinical experience and subjective opinion of the speaker.

## Introduction

As part of the 2020 Immunotherapy Bridge virtual congress (December 2nd–3rd, Italy), the Great Debate session featured counterpoint views from leading experts on three clinical questions in immunotherapy today. The first of these was whether antitumoral vaccination is still a treatment option. Despite two decades and many clinical trials of cancer vaccines, there remains only a single approved therapy, sipuleucel-T for metastatic castration-resistant prostate cancer. New approaches with improved formulations including multiple antigen vaccines and rational combinations with other therapies, including checkpoint blockade may be required. In particular, a paradigm shift from therapeutic vaccination to preventative vaccination in earlier-stage patients may be needed.

The second topic debated whether anti-programmed death (PD)-1/PD-ligand (L)1 blockade should be the backbone for immunotherapy combination. PD-1/PD-L1 is a critical target that theoretically may improve the effectiveness of any treatment that targets tumor antigen-specific immunity. Anti-PD-1/PD-L1 inhibitors are also well tolerated so can be given in combination with many types of therapy without excess toxicity. Several PD-1-based immunotherapy combinations have shown clinical activity. However, other immunotherapies have shown efficacy without the need for PD-1 blockade. An undue focus on PD-1/PD-L1 inhibition as the backbone for immunotherapy may result in combinations that are no more effective than PD-1 monotherapy. Finally, the use of innovative study designs and surrogate endpoints was considered from both an academic and industry perspective.

For each topic, two experts presented the argument and counter-argument in support of two different points of view. As with previous Bridge congresses, the debates were assigned by meeting Chairs and positions taken by experts during the debates may not have necessarily reflected their respective personal view. The views summarised in this article are based on available evidence but may reflect personal interpretation of these data, clinical experience and subjective opinion of the speaker. These perspectives are not intended to be a rigorous assessment of the topic and associated data but rather reflect two possible viewpoints and so provide the opportunity to consider different opinions. The virtual audience were asked to vote on which view they most supported both before and after the debate. Discussion of these important topics are summarised in this report.

## Antitumoral vaccination is still an option: yes or no?

### Lisa H. Butterfield: yes

Antigen presentation is the initiating event for the tumor immunity cycle. Tumor cells release cancer antigens which have to be processed and presented in order to prime and activate an effective antitumor immune response. However, tumor cells alone are poor antigen-presenting cells. Antigen-presenting cells, such as dendritic cells (DCs), are key to the initiation of the entire tumor immunity cycle.

In the USA, sipuleucel-T, an autologous antigen-presenting cell vaccine engineered to express a shared tumor antigen, was approved for the treatment of patients with asymptomatic or minimally symptomatic metastatic castration-resistant prostate cancer over 10 years ago. DC vaccines have been investigated in over 200 trials over a period of 20 years. However, we appear to have reached a ceiling effect with at best 5–10% of patients with late-stage disease achieving complete and partial responses in some trials, while in other studies no responses were seen.

The question is why cancer vaccines have not been more effective to date? One explanation may be that cancer immuno-editing involves three stages; elimination, equilibrium and escape. Natural immune responses may have already eliminated the ‘easy’ tumor cell targets leaving immuno-edited and very challenging tumor cells to be targeted by vaccines. Another reason is that endogenous T cells may be exhausted from chronic antigen stimulation.

Vaccines have been shown to initiate de novo responses to new tumor-specific antigens, can amplify an existing tumor-specific T-cell response, and can also increase the breadth and diversity of tumor-specific T-cell responses. This latter point is important because it has been shown that the higher the number of antigens that elicit a response, the better the survival of patients.

Another issue is that vaccines are complex with many components, including the antigen source, an adjuvant to render the antigen immunogenic, a vector for delivery, and mode of administration. Vaccine platforms that are immunogenic, safe and feasible include peptides, viruses, DNA, DCs, and tumor cells. However, with DC vaccines for example, we still do not know the most effective way to load the vaccine with antigen or how to best deliver the vaccine to the patient.

One new option that may offer improved outcomes is neoantigen vaccines. Neoantigens have emerged as targets of effective tumor-directed T cell responses. Increased neoantigen load is associated with improved patient outcomes. Three clinical trials of neoantigen-based vaccines in patients with melanoma, using DCs loaded with short peptides, long peptides or RNA, have shown the safety, feasibility and robust immunogenicity of this approach [1]. A crucial aspect of a vaccine targeting neoantigens is the selection of epitopes that can be presented in vivo by tumor cells or antigen-presenting cells. There is new progress in biological rules for epitope identification [2]. Optimal neoantigen delivery involving use of the most effective formulations, immune adjuvants, delivery vehicles and dosing, in combination with other therapies, will be crucial for maximum therapeutic effectiveness.

In recent studies, a DC vaccine-induced CD8+ T cell functional response was associated with improved progression-free survival (PFS) and overall survival (OS) in patients with melanoma [3]. High immune checkpoint gene expression networks correlated with inferior clinical outcomes, showing that checkpoint molecular pathways are critical for vaccine outcomes and the potential for a combined approach [4], and inducible T-cell costimulator ligand (ICOSL) on the DC vaccines was shown to be important [5]. These may be important clues for next vaccine trials. In another trial, a neoantigen vaccine generated intratumoral T cell responses in patients with glioblastoma, a tumor that generally has a low mutational burden and is considered immunologically cold [6]. Neoantigen-specific T cells from the peripheral blood were shown to migrate across the blood–brain barrier into an intracranial glioblastoma tumor. Neoantigen-targeting vaccines thus have the potential to favourably alter the immune milieu of glioblastoma.

New formulations may also provide greater success. FixVac (BNT111) is an intravenously administered liposomal RNA vaccine that targets four non-mutated, tumor-associated antigens that are prevalent in melanoma (NY-ESO-1, tyrosinase, MAGE-A3, and TPTE). FixVac, alone or in combination with anti-PD-1 checkpoint inhibition, mediated durable objective responses in checkpoint inhibitor-experienced patients with unresectable melanoma (vaccine alone: 3 partial responses and 7 with stable disease out of 25 patients; vaccine plus anti-PD-1: six partial responses out of 17 patients) [7]. Clinical responses were accompanied by the induction of strong CD4+ and CD8+ T cell immunity against the vaccine antigens. The antigen-specific cytotoxic T-cell responses in some responders were durable and reached magnitudes typically reported for adoptive T-cell therapy.

Cancer vaccines may also be more effective in earlier stage disease. Developments in imaging and other screening methods have made possible the detection of pre-malignant lesions. Therapeutic cancer vaccines based on viral antigens for the control of viral cancers have not been effective in advanced disease, which may be attributed to the immunosuppressive tumor microenvironment, but have been highly active at clearing pre-malignant lesions. Vaccines based on non-viral antigens might be similarly more effective against pre-malignant lesions of non-viral cancers, and the few completed or ongoing phase 1 and 2 clinical trials of preventive cancer vaccines have already shown clinical efficacy [8].

In summary, cancer vaccines alone can induce T cell responses and objective regressions of tumors in a small minority of late-stage cancer patients. However, greater success may be achieved using improved formulations of cells and multiple antigen vaccines (shared and neoantigens), rational combinations with other therapies, including checkpoint blockade, and vaccination of earlier-stage patients.

### Olivera J. Finn: no

A large number of vaccine clinical trials have been conducted across many human cancers with a wide range of tumor antigens having been evaluated. The extent of this work was such that, in 2009, a National Cancer Institute pilot project was carried out to prioritise some cancer antigens for further investigation based on a set of predefined and pre-weighted objective criteria such as therapeutic function, immunogenicity, specificity, tumor stem cell expression, etc. [9]. However, despite studies conducted with many different antigens and delivery systems and across all different cancer types, the results obtained were not very promising. A meta-analysis of therapeutic cancer vaccine trials conducted between 1999 and 2015 showed a large number of phase 2 studies but very few progressing to phase 3 and no approved vaccines, further illustrating the disappointing results [10]. Nothing appears to have significantly changed in the therapeutic vaccine field since 2014. Three of the most recently published phase 3 vaccine trials reported by now familiar outcomes, with induction of an immune response to some degree but no significant improvement in progression-free survival (PFS) or overall survival (OS) [11–13]

Despite these disappointments, there is an ongoing expectation that the greater understanding that these trials have provided about the immune response in the setting of cancer may point the way to improvement in the vaccine design for better outcomes. For example, a push–pull approach has been suggested by which various strategies are combined to overcome mechanisms of tumor immune escape [14]. In the initial push, immunogenicity of tumor antigens could be increased by epitope enhancement to improve their binding affinity to major histocompatibility complex (MHC) molecules. Cytokines, costimulatory molecules and other adjuvants could be used to strengthen the anti-tumor T cell response. In the effector (push) phase, multiple immunosuppressive mechanisms can be targeted by blocking or depleting regulatory cells or inhibiting regulatory molecules, e.g., by using cytotoxic T-lymphocyte-associated antigen (CTLA)-4 or PD-1 checkpoint inhibitors. However, the additional treatments suggested by this approach may have high toxicity and may also be very expensive.

In summary and justifying the “No”, therapeutic vaccination as cancer monotherapy does not appear to be a feasible treatment option. It may possibly be more effective in combination with other immunotherapies, although financial toxicity and the resulting lack of broad applicability may limit its use. It is also likely to give only a marginal improvement in clinical outcome.

A paradigm shift in cancer vaccine development is needed that involves not a change in design but a change from a therapeutic vaccination approach to a preventative one. Vaccines that target shared tumor-associated antigens that are expressed on premalignant lesions and not just on invasive cancer could prevent progression to cancer. Showing vaccine efficacy in this setting could lead eventually to vaccination of patients at risk in the absence of any disease. This may be the best way forward. The hypothesis in support of this is that preventative cancer vaccines will be more effective than therapeutic vaccines at inducing antitumor immunity because the immune system is not as suppressed in the premalignant state as it is in cancer.

**Fig. 1:**
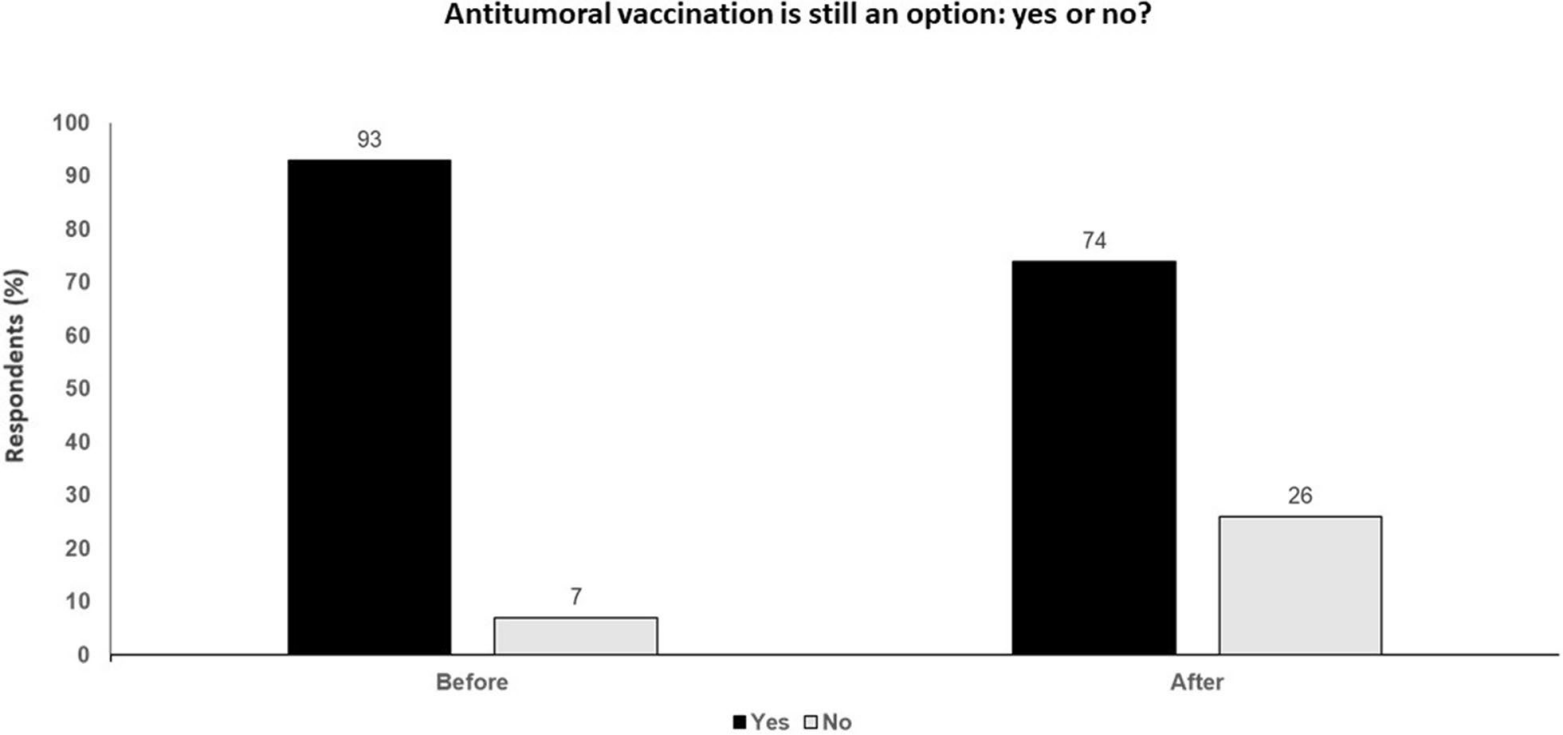
Antitumoral vaccination is still an option: yes or no? Audience response before and after debate

This new approach has already been tested with some success. In patients without cancer but with a history of premalignant lesions (advanced colonic adenomas), a vaccine based on the tumor-associated antigen MUC1 was 30 to 40 times more immunogenic than the same vaccine in previous trials in cancer [15]. Responders had high levels of anti-MUC1 immunoglobulin G and strong immune memory. Lack of response was correlated with high pre-vaccination levels of circulating myeloid-derived suppressor cells (MDSCs). This feasibility study has led to a randomized, placebo-controlled, efficacy trial of the MUC1 vaccine for colon cancer prevention, which has shown promising results, with a significant reduction in polyp recurrence (Fig. 1).

## Key points

1. Cancer vaccines have shown reliable induction of antitumor immunity and rare objective clinical responses.
2. Recent advances in DC vaccine biology, antigen selection and rational combinations may lead to greater success in the next generation of cancer vaccines.
3. Therapeutic vaccines based on a wide variety of antigens, adjuvants and delivery system have had across the board low immunogenicity and no clinical efficacy.
4. Therapeutic vaccine trials have shown a large number of immunosuppressive mechanisms in the cancer patient that lower vaccine efficacy, which can be overcome with combination therapies but with additional clinical and financial toxicity.
5. Vaccines given in the setting of pre-cancer (preventatively) are expected to be more immunogenic and more economical public health approach to the ongoing cancer pandemic.

## Is anti-PD-1/PD-L1 a backbone for immunotherapy combination: yes or no?

### Ryan J. Sullivan: yes

The development of immunotherapy over the past decade has been a remarkable achievement, with seven FDA-approved immune checkpoint inhibitors, six of which are anti-PD-1/PD-L1 antibodies. These have been approved in an increasing number of indications. In recent years, there has been an increasing focus on PD-1/PD-L1 antibodies in combination with various other therapies. Approved combinations include anti-PD-1/PD-L1 agents with ipilimumab, various cytotoxic chemotherapy regimens, bevacizumab, axitinib, lenvatinib, and vemurafenib plus cobimetinib. Combination-based approaches represent both the present and the future and it is clear that PD-1/PD-L1 antibodies are the primary backbone of these regimens.

Anti-PD-1/PD-L1 agents offer the potential for a combination approach. Firstly, they are not associated with substantial toxicity, with generally tolerable side effects meaning they, in general, can be combined with other agents without severe toxicity problems. Secondly, mechanisms of resistance to immunotherapy include alternative immune checkpoint expression (e.g. TIM3, LAG3), insufficient tumor mutation burden or neoantigens to trigger an effective immune response, insufficient priming, inadequate T cell recruitment and infiltration, the presence of regulatory T cells and/or tumor-associated macrophages, including MDSCs, that coordinate an immunosuppressive tumor microenvironment, or loss of tumor antigen-presenting machinery/loss of interferon signalling. Importantly, in every single one of these scenarios, if resistance was relieved by an alternative approach, anti-PD-1/PDL-1 inhibition should theoretically allow for a more robust immune response.

Studies have shown improved outcomes with anti-PD-1/PD-L1 based combinations versus single agent therapy. One example in melanoma in which a combination approach appears to be warranted is pembrolizumab with the multitarget tyrosine kinase inhibitor lenvatinib. In a phase 1b/2 study, pembrolizumab plus lenvatinib showed clinical activity in heavily pre-treated patients with advanced melanoma and confirmed progression on a PD-1/L1 inhibitor, including those who progressed on combined anti-PD-1/L1 plus anti-CTLA-4 therapy [16]. The confirmed overall response rate (ORR) was 21.4% in all 103 patients and 31.0% among patients with progression on prior anti-PD-1/L1 plus anti-CTLA-4. Median duration of response (DOR) was 6.3 months and 73% of patients had an estimated DOR ≥ 6 months. Grade 3–5 adverse events occurred in 45% of patients but only 8.0% discontinued lenvatinib and/or pembrolizumab as a result, suggesting toxicity of the combination can be mitigated. These results compare favourably with other data on lenvatinib as singe-agent therapy or combined with chemotherapy in melanoma, where lower response rates were observed [17, 18].

Another example is provided by the use of toripalimab, an anti-PD-1 antibody, in combination with the tyrosine kinase inhibitor axitinib. In 29 treatment-naïve mucosal melanoma patients, a high ORR of 48.3% and a disease control rate (DCR) of 86.2% were observed [19]. Median PFS was 7.5 months and median OS was 20.7 months. Treatment was tolerated with no dose-limiting toxicities or treatment-related deaths and only one patient discontinuing treatment due to an adverse event.

Other data also support the place of anti-PD-1/PD-L1 antibodies as the backbone of combination therapy. In a recently reported study, 70 patients with advanced melanoma who had progressed on anti-PD-1 monotherapy were treated with pembrolizumab and low-dose ipilimumab (1 mg/kg IV Q3 weeks); a response rate of 30% was achieved, higher than might be anticipated with single-agent ipilimumab [20]. Additionally, the median PFS was 4.7 months and 6-month PFS rate was 45%. Grade 3–4 treatment-related adverse events occurred in 21% of patients. In another study, a retrospective, multicentre analysis of over 300 patients, ipilimumab plus anti-PD-1 antibody in patients who progressed on previous anti-PD-1 therapy had a higher response rate (31% versus 12%) and longer PFS and OS than ipilimumab alone [21]. High-grade toxicity was similar in patients on the combination and ipilimumab monotherapy (≥ grade 3: 30% versus 34%). Thus, both prospective and retrospective data support the use of anti-PD-1 plus anti-CTLA-4 as second-line treatment.

In summary, anti-PD-1/PD-L1 inhibitors are well tolerated and can be given in combination with many types of therapy. PD-1/PD-L1 is a critical target that in theory should improve the effectiveness of any treatment that targets tumor antigen specific immunity. A number of combinations have shown anti-tumor activity sufficient to justify regulatory approval and have cemented the concept that anti-PD-1/PD-L1 inhibition should be the backbone of immunotherapy combinations.

### Joshua Brody: no

PD-1 blockade is a huge advance for cancer. However, despite this, many common tumor types do not respond well to anti-PD-1 antibodies, with response rates of around 5% across prostate, breast, lung, colorectal and uterine cancers and 5–8% in leukaemia and non-Hodgkins’ lymphoma (NHL).

Moreover, there are immunotherapies that provide potent clinical activity without the need for PD-1 blockade. For example, chimeric antigen receptor (CAR)-T cells can induce profound and long-lasting responses, as shown with axicabtagene ciloleucel in patients with refractory large B-cell lymphoma [22]. In theory, CAR-T cell therapy should benefit from being combined with PD-1 blockade, given T cells are activated and PD-L1 upregulated in the tumor. However, the treatment is effective even without PD-1 blockade. Another example is the use of T cell bispecific monoclonal antibodies, such as glofitamab (formerly known as CD20-TCB), which consists of antigen-binding regions for CD20 (on B cells) and CD3 (on T cells) and offers the potential for increased tumor antigen avidity, rapid T-cell activation, and enhanced tumor cell killing. This has shown a profound effect in lymphoma, with response rates of nearly 70% in low-grade lymphoma and 50% in aggressive lymphoma in heavily pre-treated patients, with many complete and long-lasting remissions [23].

An in situ vaccine that combined Flt3L, radiotherapy, and a toll-like receptor (TLR)-3 agonist, was used to recruit, antigen-load and activate intratumoral, cross-presenting DCs. In patients with advanced stage indolent NHL, this approach induced anti-tumor CD8+ T cell responses and systemic (abscopal) tumor regressions [24]. Again, this approach appears to be effective without the need to be combined with PD-1 blockade.

The concept of PD-1 blockade as the backbone of combination therapy implies combining other agents with PD-1 inhibitors without the additional treatment necessarily having shown efficacy as monotherapy. However, novel immunotherapies should first demonstrate efficacy as single agents. One example of this is IDO inhibitor epacadostat, which failed to show any objective responses as monotherapy in a first-in-human phase I study in patients with advanced solid malignancies [25]. A single-arm phase 1/2 study of epacadostat and pembrolizumab in 64 patients with advanced melanoma showed encouraging results, with an ORR of 56% and median PFS of 12.4 months [26]. However, in a subsequent phase 3 trial, epacadostat plus pembrolizumab did not improve PFS or OS versus pembrolizumab monotherapy in patients with unresectable or metastatic melanoma [27]. This suggests that simply adding treatments to a PD-1 inhibitor backbone may not be the best approach. The use of ineffective monotherapies in combination with PD-1 blockade can lead to ‘false-positive’ phase 2 combination studies and the subsequent waste of resource and effort of large-scale phase 3 studies that subsequently fail to meet their primary endpoint.

**Fig. 2:**
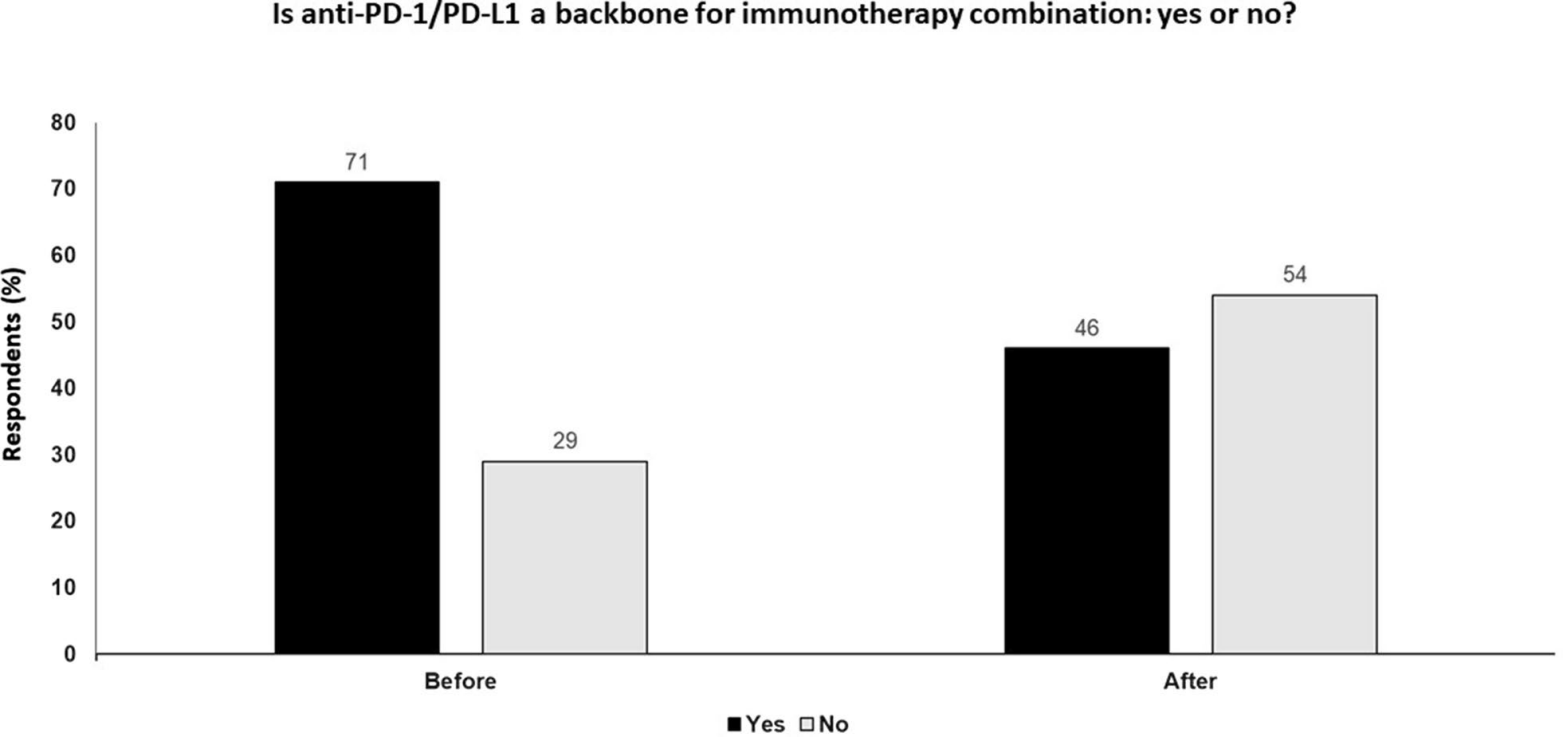
Is anti-PD-1/PD-L1 a backbone for immunotherapy combination: yes or no? Audience response before and after debate

However, anti-PD-1 therapy can be usefully combined with some therapies that induce a monotherapy response and adaptive resistance. This was shown by the in situ vaccine that induced PD-1-positive T cells, and rendered murine tumors newly responsive to PD-1 blockade [24]. This is now being investigated in a phase 3 trial of the Flt3L-primed in situ vaccine in combination with pembrolizumab (Fig. 2).

## Key points

1. Checkpoint blockade is a huge advance for cancer and still leaves an unmet need.
2. Immunotherapies clearly *can* eliminate tumors without PD-1 blockade, e.g. CAR-T, bispecific antibodies (e.g. CD3 × CD20), and in situ vaccines.
3. Small trials of PD-1 blockade *combined* with novel immunotherapies have given unrealistic expectations about the combinations’ efficacy, prompting large, randomized trials which soak up limited resources and drive the field in, potentially, unproductive directions, detracting from alternative approaches.
4. It is critical that novel immunotherapies first demonstrate single agent efficacy before being used in combination with PD-1 blockade so that the *most* promising combinations can be moved forwards.

## Do we need an innovation in study design and is overall survival the endpoint? Points of view from academia and industry

### Francesco Perrone: view from academia

The approval of new drugs by regulatory agencies is primarily based on the quality of the evidence, and the value and affordability of the new treatment. This is consistent and does not change according to the type of drug, the type of disease and the statistical methodology applied in registrative clinical trials. However, some recent trends requiring attention are (i) the use of innovative study designs, (ii) the progressive weakening of the evidence required for approval and (iii) the ever-increasing cost of new drugs.

The experience with SARS-Cov2 vaccine development and approval has shown that the whole process of new drug development can be realised in a much shorter timeframe, with stages that were previously sequential now occurring in parallel. In the future, this experience may help to inform drug development in other areas such as cancer.

At some point in the clinical development programme, randomized trials are important in order to ensure high quality evidence. However, this does not negate the need for innovative study design. Types of design that have been increasingly popular over the past two decades include basket, umbrella and platform trials [28]. Basket trials involve a targeted therapy being evaluated in multiple diseases with a common molecular aberration, while umbrella trials evaluate multiple targeted therapies in a single disease stratified into molecular subgroups. Platform trials evaluate several interventions against a common control group, can drop arms early or add extra arms, and can be continued indefinitely.

With regard to quality of evidence, it is also important not to rely on non-validated surrogate endpoints as the sole measure of efficacy. A valid surrogate endpoint is assessed much earlier than the true endpoint, can be easily and reliably measured, is able to predict what will happen with the true endpoint, and plays a causal role in determination of the true endpoint. These characteristics need to be demonstrated; however large-scale randomized controlled trials, or even meta-analyses of such trials, which measure both surrogate and true endpoints are needed for validation. Moreover, validation is disease-specific, stage-specific and treatment or treatment class-specific. As such, it is a chimera that surrogate endpoints can be validated for new treatment options.

An analysis of trial-level validation studies, indeed, showed that most surrogate end points in oncology had low correlations with survival, especially in the metastatic setting [29]. While surrogate endpoints become more and more popular, we should not forget that they often inflate the true clinical benefit. In patients with non-metastatic castration-resistant prostate cancer, for instance, apalutamide, darolutamide and enzalutamide each showed a median metastasis-free survival (MFS) benefit of around 22–24 months in three separate randomized, placebo-controlled, phase 3 trials, with hazard ratios of 0.28–0.41. However, when 4-year OS rates were assessed, hazard ratios were in the range of 0.69–0.78 and numbers needed-to-treat were much higher (14–28 versus 4–6). Thus, the use of MFS as a surrogate endpoint indicated an overestimated clinical benefit, as compared to true effect seen on OS.

Surrogate endpoints also allow accelerated approval of new treatments. A survey of accelerated approvals for cancer treatments by the US FDA reported that all (93 indications) were based on a surrogate primary endpoint. Of these, only 15 (16%) showed a subsequent OS benefit [30]. This compared with 78% of all European Medicines Agency cancer drug approvals from 2009 to 20,913 based on a surrogate endpoint, of which 51% showed a subsequent OS or quality of life benefit [31]. Accelerated approval may therefore be obtained with limited information available regarding the benefits of novel cancer treatments at market entry; this, however, may lower the confidence of regulators, payers and, importantly, also clinicians.

Both ASCO and ESMO have attempted to develop a framework to assess the value of cancer treatments. However, this has proved challenging. What is clear is that novel cancer treatments are expensive and that the high costs of many of these treatments is unsustainable and results in financial toxicity and lack of access. Affordability, accessibility and sustainability are the main focus of the new EU pharmaceutical strategy.

### John Goldberg: view from industry

OS is the gold standard but other endpoints may be relevant to patients. The goal of cancer drug development is safe and effective treatments for patients who need them. More rapid approval of new drugs, with appropriate assurance of safety, may be better, especially if there are no current treatments. In this situation, endpoints other than OS may have to be considered. If treatment already exists, but is perhaps problematic or less efficacious, OS may be more critical. If new treatments appear to be safer, more tolerable or less burdensome but with similar efficacy, safety and/or quality of life endpoints may be relevant. Thus, the best strategy depends on the clinical situation for the indication under consideration, and requires buy-in from stakeholders (e.g., sponsor, investigators, regulators, and patients). Planning in early development can help guide us to the right endpoints.

Early drug development involves identifying the correct dose. The 3 + 3 design is the most common choice for phase I dose-escalation oncology trials. However, the 3 + 3 design has now been augmented by the modified toxicity probability interval (mTPI) design, which may be a safer and more reliable method. The key here is to identify the correct dose and then go to the next step. Dose expansion is usually the next stage after dose escalation. There is a need to make an educated guess about the likely most relevant indications. Enrolling patients will increase safety, biomarker and efficacy data after typical basket escalation. Many studies will have expansion cohorts after escalation. This stage is difficult to get right without any clinical data but important to plan. Expansion cohorts allow better estimates of indications to select, size of possible phase 2 trials, designs, endpoints and likely magnitude of change. These data combined with knowledge of indications refines the next steps. If the data in expansion is meaningful enough for patients to generate a Break-Through Designation, approval may be possible based on an amended phase 1 study from an expansion with ORR (e.g., as with pembrolizumab). This is not easy, and with more and more immuno-oncology trials, such striking data in expansion is likely to be the exception. Once there is a robust understanding of the benefit rate in an indication, registrational trials can be designed.

If there is no standard of care for the disease, for example, after progression on standard of care immunotherapy, then demonstrating an endpoint of ORR that is meaningful, and durable, could be an appropriate goal for a development program. Meaningful ORR will depend on the indication. For example, if there are no other acceptable options, ~ 20% may be sufficient. However, for an ORR to be meaningful it needs to be durable, with a substantial portion of patients maintaining their response 6 to 12 months after it is first noted.

In an analysis of 13 randomized, active-controlled trials of immunotherapies to assess ORR and PFS as surrogate endpoints of OS, patients with an ORR had longer PFS and OS than non-responders [32]. However, the trial-level and individual-level associations were weak between ORR or PFS and OS. This shows we need to assess use of surrogate endpoints on a case-by-case basis.

PFS is an attractive endpoint and is meaningful for patients. For clinical trial design, using PFS can overcome the problem of the impact of immuno-oncology therapy after trial participation on OS in studies with randomised controlled design (i.e., patients on placebo switching to immunotherapy). However, does PFS correlate with OS? One option that has been proposed is to consider time from randomization to second disease progression (PFS2). A meta-analysis showed a positive correlation for PFS2 and OS was found for all 15 studies that reported both endpoints, supporting the use of PFS2 to measure long-term clinical benefit when OS cannot be assessed [33]. However, in practice time to second progression may be difficult to monitor in a clinical trial.

Complete pathological response (pCR) makes a lot of sense as a surrogate endpoint for early breast cancer where the goal is to warn of a later relapse and the endpoint correlates with ultimate survival. Accelerated approval might be granted on the basis of pCR rate, but there is still a need to show an effect on OS so planning for confirmatory effort must be started early. Drug developers also welcome meaningful approaches to pCR in other diseases. Circulating tumor DNA (ctDNA) and other residual disease markers are of significant interest and are being pioneered in haematological diseases. However, it is not yet clear whether they will be validated as an endpoint for immunotherapy trials.

**Fig. 3:**
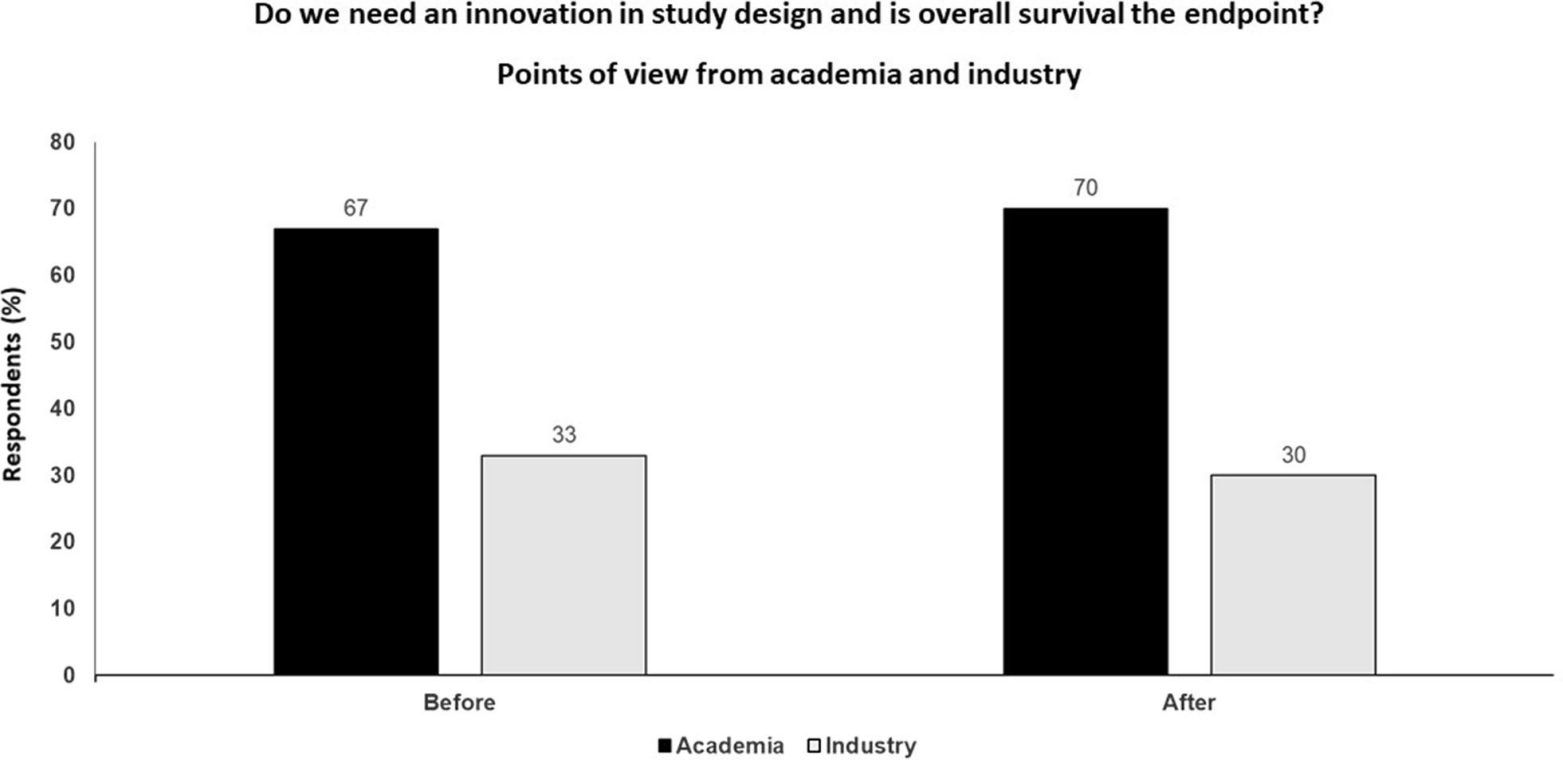
Do we need an innovation in study design and is overall survival the endpoint? Points of view from academia and industry. Audience response before and after debate

OS is the goal that we all want, assuming the extra time offers a good quality of life and is of significant and meaningful duration (i.e., more than a few weeks). Limitations of OS include the longer time to reach study conclusion in some diseases and confounding by control patients going onto successful treatments after completing study participation. Whether a drug should be approved/continue to be approved if it has not shown an OS benefit may depend on the situation. Surrogate endpoints such as PFS, ORR and quality-of-life measures all have a role in improving the lives of cancer patients and may help evaluate therapies and get them to patients faster. If these metrics are significant, OS may not always be needed for approval. If these endpoints are not significant, a lack of OS benefit is tantamount to saying the new therapy may not be effective in this situation. The only biomarkers about which patients care are response and survival, as long as survival is associated with good quality of life (Fig. 3).

## Key points

1. Non-validated surrogate endpoints should not be relied upon as the sole measure of efficacy. Validation of surrogate endpoints is disease-specific, stage-specific and treatment or treatment class-specific.
2. Most surrogate end points have low correlations with survival, especially in the metastatic setting, and often inflate the true clinical benefit.
3. Surrogate endpoints allow accelerated approval of new treatments; this, however, may lower the confidence of regulators, payers and, importantly, also clinicians.
4. Rapid approval of new drugs, with appropriate assurance of safety, may be important, especially if there are no effective treatments available.
5. Surrogate endpoints such as PFS, ORR and quality-of-life measures all have a role in improving the lives of cancer patients and may help evaluate therapies and get them to patients faster.

## Conclusions

Over the last few years, extensive research has improved our understanding of tumor immunology and enabled the development of novel treatments that can harness the patient’s immune system and prevent immune escape. Through numerous clinical trials and real-world experience, a large amount of evidence of the potential for long-term survival with immunotherapy agents has been accumulated in various types of malignancy, starting from melanoma and extending to other tumors. The results of these studies have also highlighted a number of recurring observations with immuno-oncology agents, including their potential for clinical application across a broad patient population. In these Great Debate sessions, three topical clinical issues in immunotherapy were discussed. Given the constraints of the format and the intended nature of the session, each presentation was not intended as a rigorous assessment of the field but rather provided an opportunity to highlight some important areas of debate within immunotherapy. We hope that these discussions can focus attention on these issues, encouraging further research on these important topics.

## Data Availability

Not applicable.

## Abbreviations

CAR: Chimeric antigen receptor
ctDNA: Circulating tumor DNA
CTLA-4: Cytotoxic T-lymphocyte-associated antigen-4
DC: Dendritic cell
DCR: Disease control rate
DOR: Duration of response
ICOSL: Inducible T-cell costimulator ligand
MDSC: Myeloid-derived suppressor cell
MFS: Metastasis-free survival
MHC: Major histocompatibility complex
mTPI: Modified toxicity probability interval
NHL: Non-Hodgkins’ lymphoma
ORR: Overall response rate
OS: Overall survival
pCR: Pathological complete response
PD-1: Programmed death-1
PD-L1: Programmed death ligand-1
PFS: Progression-free survival
TLR: Toll-like receptor
